# Learning psychological research and statistical concepts using retrieval-based practice

**DOI:** 10.3389/fpsyg.2015.01484

**Published:** 2015-10-05

**Authors:** Stephen Wee Hun Lim, Gavin Jun Peng Ng, Gabriel Qi Hao Wong

**Affiliations:** Department of Psychology, Faculty of Arts and Social Sciences, National University of Singapore, Singapore, Singapore

**Keywords:** retrieval-based learning, testing effect, research methods pedagogy, teaching of psychology, experimental education

## Abstract

Research methods and statistics are an indispensable subject in the undergraduate psychology curriculum, but there are challenges associated with engaging students in it, such as making learning durable. Here we hypothesized that retrieval-based learning promotes long-term retention of statistical knowledge in psychology. Participants either studied the educational material in four consecutive periods, or studied it just once and practiced retrieving the information in the subsequent three periods, and then took a final test through which their learning was assessed. Whereas repeated studying yielded better test performance when the final test was immediately administered, repeated practice yielded better performance when the test was administered a week after. The data suggest that retrieval practice enhanced the learning—produced better long-term retention—of statistical knowledge in psychology than did repeated studying.

## Introduction

Research methods and statistics are integral to an education in psychology. Ninety-eight percentage of undergraduate psychology programs in North America mandate their students to take at least one methodology class (Stoloff et al., 2009). Psychology graduates who have undergone statistical training acquire critical reasoning skills, distinguishing them from those who have not taken statistics or research methodology classes (Lehman and Nisbett, 1990; Lawson, 1999). Yet, statistics classes can be a source of anxiety (Tremblay et al., 2000) and a dreaded component of the undergraduate psychology curriculum (Conners et al., 1998).

Conners et al. (1998) enumerated four unique challenges for the teaching and learning of undergraduate statistics specifically relating to (a) motivating students, (b) math anxiety (an emotional state of dread toward future math-related activities; see Hembree, 1990), (c) performance extremes and, finally, (d) making learning durable which is of particular interest to the present research. Many educators have noted that students remember very little of what they have previously learned in statistics. One reason is that statistics is akin to a new language, comprising of unique vocabulary and syntax. Lalonde and Gardner (1993) showed that learning statistics is analogous to learning a second language, and argued that it is difficult for students to achieve and maintain fluency with limited exposure. The goal is to discover ways to enhance the learning—increase the retention—of statistical knowledge.

Learning has traditionally been equated to the encoding process through which knowledge is acquired whereas retrieval, often through testing, is viewed as merely a means to judge the extent of prior learning (see, e.g., Karpicke and Roediger, 2008). A fast-growing body of research reveals, however, that retrieval actually aids the retention of previously learned information (e.g., Chan and McDermott, 2007; Roediger and Butler, 2011). This phenomenon of improved knowledge recall afforded by retrieval episodes has been referred to as the testing effect (e.g., Carrier and Pashler, 1992), test-enhanced learning (e.g., Roediger and Karpicke, 2006) and, more recently, retrieval-based learning (e.g., Karpicke, 2012).

In the standard retrieval-based learning paradigm, learners either studied educational materials repeatedly, or studied and then practiced retrieving the materials, before taking a final test to assess their learning. In Roediger and Karpicke (2006; Experiment 2), students either studied a prose passage once and underwent three free recall tests about the material, studied the passage three times and took one test, or basically studied the passage four times. They then took a final retention test either 5 min or 1 week later. Traditionally, massed studying produces short-term knowledge retention benefits (see, e.g., Balota et al., 1989). Unsurprisingly, Roediger and Karpicke (2006) found that students who studied the material repeatedly performed better when the retention test was administered immediately. The crucial finding, however, was that students who practiced retrieving performed better when the test was administered 1 week later, implicating the positive effects of retrieval practice on longer-term retention of educationally relevant knowledge (see, also, Gates, 1917).

Lyle and Crawford (2011) implemented the idea of test-enhanced learning in a statistics for psychology course, and found that the student cohort that underwent testing after each lecture eventually obtained higher exam scores than did the cohort which was not tested. While the data imply that testing is advantageous for learning, this advantage is attributable to such reasons as the students in the tested group were simply more motivated to attend lectures—and paid more attention during lectures, since those end-lecture tests were formally graded and students would have taken them seriously. In other words, it is unclear whether the advantage observed was simply due to the fact that the tested cohort basically attended (to) lectures more faithfully than did the untested cohort, rather than due to the prowess of test-enhanced learning *per se*.

### The Present Study

Our goal was to illuminate the effects of retrieval-based practice in learning psychological research and statistical concepts under an experimental setting. In line with extant empirical work (e.g., Roediger and Karpicke, 2006; Toppino and Cohen, 2009; Coppens et al., 2011; Kornell et al., 2011) which showed that retrieval-based practice enhances long-term learning, we made two predictions. First, repeated studying—relative to retrieval-based practice—would improve performance when a final test was immediately administered. In contrast, and more important, retrieval-based practice would lead to superior performance in the final delayed test administered after a week.

## Materials and Methods

### Participants

Sixty-five psychology undergraduates at the National University of Singapore participated for either course credit or a monetary incentive ($10 for an hour of participation). Those who have taken a research methods and statistics course in psychology were excluded from participation. This research was conducted with the appropriate ethics review board approval by the National University of Singapore, and participants have granted their written informed consent.

### Materials

A prose passage on the topic of hypothesis testing was developed based on the contents of a textbook chapter by Aron et al. (2009). The passage comprised of concepts in hypothesis testing, central tendency, and decision errors; it contained 361 words, and was decomposable into 26 idea units for scoring purposes.

### Design

A 2 × 2 fully-between design was employed: Participants were randomly assigned to one of two learning conditions: (a) repeated study (SSSS; 36 participants) or (b) retrieval-practice (SRRR; 29 participants). Within each learning condition, about half the participants were assigned to take a final recall test after a 5-min retention interval, whereas the remaining participants took the same recall test after a 1-week retention interval. The dependent variable was proportion of idea units recalled.

### Procedure

Participants underwent two sessions. During Phase 1, participants in the repeated study condition studied the passage for four 5-min periods, whereas those in the retrieval practice condition first studied the passage in the first 5-min period and practiced retrieving what they studied in the next three periods, writing down as much material as they could remember from the passage. Participants solved multiplication problems for 2 min in between periods and 5 min at the end of Phase 1. Phase 2 comprised of a 10-min period, during which the final recall test was administered either after 5 min or 1 week later. Participants were asked to recall as much knowledge as they could from the passage administered during Phase 1.

## Results and Discussion

**FIGURE 1:**
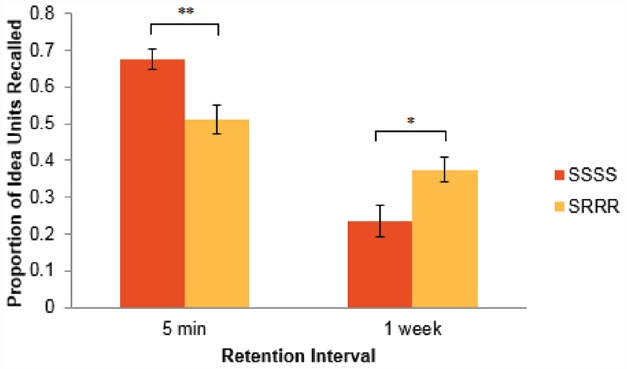
**Proportion of idea units recalled across learning condition (***SSSS*** versus ***SRRR***) and retention interval (5-min versus 1-week).** Error bars denote standard errors. *p < .05; **p < .01.

Participants were awarded one point for correctly recalling each of the 26 idea units. The data were then submitted to a 2 × 2 analysis of variance (ANOVA). All assumptions for ANOVA, including independence, normality, and homogeneity of variances, were met. A significant interaction between learning condition and retention interval emerged, *F*(1,61) = 17.87, *p* < .001. Post hoc analyses showed that in the 5-min retention interval condition, repeated study led to a higher proportion of idea units being recalled (*M* = 0.675, SD = 0.125) than did retrieval practice (*M* = 0.512, SD = 0.143), *t*(31) = 3.47, *p* = .002. In contrast, in the 1-week retention interval condition, retrieval practice led to better recall performance (*M* = 0.375, SD = 0.130) than did repeated studying (*M* = 0.236, SD = 0.173), *t*(30) = 2.58, *p* = .015. These findings appear summarily in Figure 1.

The data supported both of our predictions. While repeated studying, relative to retrieval-based practice, improved recall performance when a final test was immediately administered, retrieval-based practice led to better performance than did repeated studying when the final test was administered after a week. It is worth emphasizing that even though learners who underwent repeated studying read the passage an average of 8.71 times while those who underwent retrieval practice did so only 2.44 times, the latter group was able to recall significantly more idea units after a week has lapsed. Retrieval practice enhances the retention of verbatim knowledge in psychological research and statistical concepts. We have now begun investigating in our Lab whether, and to what extent, retrieval-based learning enhances analogical problem solving — the transfer of previously acquired knowledge or solutions from one context to another — involving psychological research and statistical concepts.

### Conflict of Interest Statement

The authors declare that the research was conducted in the absence of any commercial or financial relationships that could be construed as a potential conflict of interest.
